# Supplementary material for: Chitosan-olive oil microparticles for phenylethyl isothiocyanate delivery: Optimal formulation
Source: PLoS One. 2021 May 6;16(5):e0248257. doi: 10.1371/journal.pone.0248257 (PMC8101728; doi:10.1371/journal.pone.0248257)
Supplement: S1 Appendix — (DOCX) [file pone.0248257.s001.docx]

**S1 Appendix**

Raw data and full statistical analysis of the 3-level (3^2^) factorial experimental design.

**Data matrix**

| *Run* | X_A_ | X_B_ | *Z-average diameter (nm)* | *PDI* | *Zeta-potential (mV)* | *EE (%)* |
| --- | --- | --- | --- | --- | --- | --- |
|  |  |  |  |  |  |  |
| 1 | 2.5 | 0.75 | 746.0 | 0.269 | 12.6 | 99.604 |
| 2 | 1.25 | 0.75 | 659.0 | 0.267 | 21.4 | 99.624 |
| 3 | 0 | 0.25 | 893.0 | 0.677 | 34.5 | 98.155 |
| 4 | 1.25 | 1.25 | 653.0 | 0.266 | 14.1 | 99.753 |
| 5 | 0 | 1.25 | 2139.0 | 0.315 | 31.8 | 99.528 |
| 6 | 2.5 | 1.25 | 712.0 | 0.283 | 5.54 | 99.599 |
| 7 | 0 | 0.75 | 1355.0 | 0.464 | 37.7 | 99.4 |
| 8 | 2.5 | 0.25 | 623.0 | 0.283 | 23.7 | 99.021 |
| 9 | 1.25 | 0.25 | 629.0 | 0.283 | 31.7 | 99.004 |
| 10 | 2.5 | 0.75 | 702.0 | 0.303 | 7.99 | 99.344 |
| 11 | 1.25 | 0.75 | 665.0 | 0.279 | 20.8 | 99.582 |
| 12 | 0 | 0.25 | 686.0 | 0.583 | 37.0 | 98.277 |
| 13 | 1.25 | 1.25 | 684.0 | 0.267 | 15.4 | 99.719 |
| 14 | 0 | 1.25 | 2237.0 | 0.275 | 29.6 | 99.67 |
| 15 | 2.5 | 1.25 | 751.0 | 0.282 | 5.65 | 99.69 |
| 16 | 0 | 0.75 | 1703.0 | 0.499 | 37.8 | 99.458 |
| 17 | 2.5 | 0.25 | 680.0 | 0.267 | 20.9 | 99.2 |
| 18 | 1.25 | 0.25 | 617.0 | 0.261 | 33.8 | 98.829 |
| 19 | 2.5 | 0.75 | 738.0 | 0.29 | 11.1 | 99.438 |
| 20 | 1.25 | 0.75 | 666.0 | 0.256 | 24.2 | 99.43 |
| 21 | 0 | 0.25 | 744.0 | 0.593 | 29.6 | 98.208 |
| 22 | 1.25 | 1.25 | 694.0 | 0.262 | 10.2 | 99.491 |
| 23 | 0 | 1.25 | 2292.0 | 0.306 | 33.3 | 99.705 |
| 24 | 2.5 | 1.25 | 743.0 | 0.257 | 5.82 | 99.651 |
| 25 | 0 | 0.75 | 1250.0 | 0.497 | 37.0 | 99.456 |
| 26 | 2.5 | 0.25 | 721.0 | 0.284 | 14.9 | 99.021 |
| 27 | 1.25 | 0.25 | 628.0 | 0.281 | 31.5 | 98.276 |

**Factorial analysis of experiments for Z-average diameter**

**Initial model**

**Estimated effects for Z-average diameter (nm)**

| *Effect* | *Estimated* | *Std. Error* | *V.I.F.* |
| --- | --- | --- | --- |
| Mean | 649.148 | 76.1107 |  |
| X_A_ | -764.778 | 83.3751 | 1.0 |
| X_B_ | 520.444 | 83.3751 | 1.0 |
| X_A_^2^ | 880.556 | 144.41 | 1.0 |
| X_A,B_ | -693.833 | 102.113 | 1.0 |
| X_B_^2^ | 17.5556 | 144.41 | 1.0 |

Standard errors based on total error with 21 d.f.

**Analysis of Variance for Z-average diameter**

| *Source* | *Sum of squares* | *DF* | *Mean Square* | *F* | *P* |
| --- | --- | --- | --- | --- | --- |
| X_A_ | 2.63198E6 | 1 | 2.63198E6 | 84.14 | 0.0000 |
| X_B_ | 1.21888E6 | 1 | 1.21888E6 | 38.97 | 0.0000 |
| X_A_^2^ | 1.16307E6 | 1 | 1.16307E6 | 37.18 | 0.0000 |
| X_A,B_ | 1.44421E6 | 1 | 1.44421E6 | 46.17 | 0.0000 |
| X_B_^2^ | 462.296 | 1 | 462.296 | 0.01 | 0.9044 |
| Total Error | 656908. | 21 | 31281.3 |  |  |
| Total (corr.) | 7.11551E6 | 26 |  |  |  |

R^2^ = 90.768 %

R^2^ _adj_ = 88.5698 %

Standard error of the estimate = 176.865

Mean absolute error = 130.929

Durbin-Watson = 2.10756 (P=0.6689

Lag 1 residual autocorrelation = -0.0953037

**Recalculated model**

**Estimated effects for Z-average diameter (nm)**

| *Effect* | *Estimated* | *Std. Error* | *V.I.F.* |
| --- | --- | --- | --- |
| Mean | 655.0 | 57.6199 |  |
| X_A_ | -764.778 | 81.4868 | 1.0 |
| X_B_ | 520.444 | 81.4868 | 1.0 |
| X_A_^2^ | 880.556 | 141.139 | 1.0 |
| X_A,B_ | -693.833 | 99.8006 | 1.0 |

Standard errors based on total error with 22 d.f.

**Analysis of Variance for Z-average diameter**

| *Source* | *Sum of squares* | *DF* | *Mean Square* | *F* | *P* |
| --- | --- | --- | --- | --- | --- |
| X_A_ | 2.63198E6 | 1 | 2.63198E6 | 88.08 | 0.0000 |
| X_B_ | 1.21888E6 | 1 | 1.21888E6 | 40.79 | 0.0000 |
| X_A_^2^ | 1.16307E6 | 1 | 1.16307E6 | 38.92 | 0.0000 |
| X_A,B_ | 1.44421E6 | 1 | 1.44421E6 | 48.33 | 0.0000 |
| Total Error | 657370. | 22 | 29880.5 |  |  |
| Total (corr.) | 7.11551E6 | 26 |  |  |  |

R^2^ = 90.7615 %

R^2^ _adj_ = 89.0817 %

Standard error of the estimate = 172.86

Mean absolute error = 130.496

Durbin-Watson = 2.11364 (P=0.6842)

Lag 1 residual autocorrelation = -0.0990234

**Regression coefficients for Z-average diameter**

| *Coefficient* | *Estimated* |
| --- | --- |
| β_0_ | 566.958 |
| β_A_ | -594.056 |
| β_B_ | 1214.28 |
| β_A,A_ | 281.778 |
| β_A,B_ | -555.067 |

**Correlation Matrix for Estimated Effects**

|  |  | (1) | (2) | (3) | (4) | (5) |
| --- | --- | --- | --- | --- | --- | --- |
| (1) | Mean | 1.0000 | 0.0000 | 0.0000 | -0.8165 | 0.0000 |
| (2) | X_A_ | 0.0000 | 1.0000 | 0.0000 | 0.0000 | 0.0000 |
| (3) | X_B_ | 0.0000 | 0.0000 | 1.0000 | 0.0000 | 0.0000 |
| (4) | X_A_^2^ | -0.8165 | 0.0000 | 0.0000 | 1.0000 | 0.0000 |
| (5) | X_A,B_ | 0.0000 | 0.0000 | 0.0000 | 0.0000 | 1.0000 |

**Estimated Results for Z-average diameter**

| *Run* | *Observed values* | *Adjusted values* | *Std. Error* |
| --- | --- | --- | --- |
| 1 | 746.0 | 712.889 | 182.21 |
| 2 | 659.0 | 655.0 | 182.21 |
| 3 | 893.0 | 870.528 | 193.263 |
| 4 | 653.0 | 915.222 | 186.71 |
| 5 | 2139.0 | 2084.81 | 193.263 |
| 6 | 712.0 | 626.194 | 193.263 |
| 7 | 1355.0 | 1477.67 | 182.21 |
| 8 | 623.0 | 799.583 | 193.263 |
| 9 | 629.0 | 394.778 | 186.71 |
| 10 | 702.0 | 712.889 | 182.21 |
| 11 | 665.0 | 655.0 | 182.21 |
| 12 | 686.0 | 870.528 | 193.263 |
| 13 | 684.0 | 915.222 | 186.71 |
| 14 | 2237.0 | 2084.81 | 193.263 |
| 15 | 751.0 | 626.194 | 193.263 |
| 16 | 1703.0 | 1477.67 | 182.21 |
| 17 | 680.0 | 799.583 | 193.263 |
| 18 | 617.0 | 394.778 | 186.71 |
| 19 | 738.0 | 712.889 | 182.21 |
| 20 | 666.0 | 655.0 | 182.21 |
| 21 | 744.0 | 870.528 | 193.263 |
| 22 | 694.0 | 915.222 | 186.71 |
| 23 | 2292.0 | 2084.81 | 193.263 |
| 24 | 743.0 | 626.194 | 193.263 |
| 25 | 1250.0 | 1477.67 | 182.21 |
| 26 | 721.0 | 799.583 | 193.263 |
| 27 | 628.0 | 394.778 | 186.71 |

**Response optimisation**

Goal: minimise Z-average diameter

Optimal value = 394.063 nm

| *Factor* | *Low* | *High* | *Optimal* |
| --- | --- | --- | --- |
| X_A_ | 0.0 | 2.5 | 1.30029 |
| X_B_ | 0.25 | 1.25 | 0.25 |

**Factorial analysis of experiments for Zeta-potential**

**Initial model**

**Estimated effects for Zeta-potential (mV)**

| *Effect* | *Estimated* | *Std. Error* | *V.I.F.* |
| --- | --- | --- | --- |
| Mean | 23.0174 | 1.58987 |  |
| X_A_ | -22.2333 | 1.74161 | 1.0 |
| X_B_ | -11.7989 | 1.74161 | 1.0 |
| X_A_^2^ | 1.14444 | 3.01656 | 1.0 |
| X_A,B_ | -6.015 | 2.13303 | 1.0 |
| X_B_^2^ | -1.35222 | 3.01656 | 1.0 |

Standard errors based on total error with 21 d.f.

**Analysis of Variance for Zeta-potential**

| *Source* | *Sum of squares* | *DF* | *Mean Square* | *F* | *P* |
| --- | --- | --- | --- | --- | --- |
| X_A_ | 2224.45 | 1 | 2224.45 | 162.97 | 0.0000 |
| X_B_ | 626.462 | 1 | 626.462 | 45.90 | 0.0000 |
| X_A_^2^ | 1.96463 | 1 | 1.96463 | 0.14 | 0.7082 |
| X_A,B_ | 108.541 | 1 | 108.541 | 7.95 | 0.0103 |
| X_B_^2^ | 2.74276 | 1 | 2.74276 | 0.20 | 0.6586 |
| Total Error | 286.639 | 21 | 13.6495 |  |  |
| Total (corr.) | 3250.79 | 26 |  |  |  |

R^2^ = 91.1825 %

R^2^ _adj_ = 89.0831 %

Standard error of the estimate = 3.69452

Mean absolute error = 2.69649

Durbin-Watson = 1.72739 (P=0.3042)

Lag 1 residual autocorrelation = 0.117746

**Recalculated model**

**Estimated effects for Zeta-potential (mV)**

| *Effect* | *Estimated* | *Std. Error* | *V.I.F.* |
| --- | --- | --- | --- |
| Mean | 22.9481 | 0.68495 |  |
| X_A_ | -22.2333 | 1.67778 | 1.0 |
| X_B_ | -11.7989 | 1.67778 | 1.0 |
| X_A,B_ | -6.015 | 2.05485 | 1.0 |

Standard errors based on total error with 23 d.f.

**Analysis of Variance for Zeta-potential**

| *Source* | *Sum of squares* | *DF* | *Mean Square* | *F* | *P* |
| --- | --- | --- | --- | --- | --- |
| X_A_ | 2224.45 | 1 | 2224.45 | 175.61 | 0.0000 |
| X_B_ | 626.462 | 1 | 626.462 | 49.46 | 0.0000 |
| X_A,B_ | 108.541 | 1 | 108.541 | 8.57 | 0.0076 |
| Total Error | 291.346 | 23 | 12.6672 |  |  |
| Total (corr.) | 3250.79 | 26 |  |  |  |

R^2^ = 91.0377 %

R^2^ _adj_ = 89.8687 %

Standard error of the estimate = 3.55911

Mean absolute error = 2.73631

Durbin-Watson = 1.63406 (P=0.1942)

Lag 1 residual autocorrelation = 0.16988

**Regression coefficients for Zeta-potential**

| *Coefficient* | *Estimated* |
| --- | --- |
| β_0_ | 38.4027 |
| β_A_ | -5.28433 |
| β_B_ | -5.78389 |
| β_A,B_ | -4.812 |

**Correlation Matrix for Estimated Effects**

|  |  | (1) | (2) | (3) | (4) |
| --- | --- | --- | --- | --- | --- |
| (1) | Mean | 1.0000 | 0.0000 | 0.0000 | 0.0000 |
| (2) | X_A_ | 0.0000 | 1.0000 | 0.0000 | 0.0000 |
| (3) | X_B_ | 0.0000 | 0.0000 | 1.0000 | 0.0000 |
| (4) | X_A,B_ | 0.0000 | 0.0000 | 0.0000 | 1.0000 |

**Estimated Results for Zeta-potential**

| *Run* | *Observed values* | *Adjusted values* | *Std. Error* |
| --- | --- | --- | --- |
| 1 | 12.6 | 11.8315 | 3.72023 |
| 2 | 21.4 | 22.9481 | 3.62442 |
| 3 | 34.5 | 36.9568 | 3.94962 |
| 4 | 14.1 | 17.0487 | 3.72023 |
| 5 | 31.8 | 31.1729 | 3.94962 |
| 6 | 5.54 | 2.92454 | 3.94962 |
| 7 | 37.7 | 34.0648 | 3.72023 |
| 8 | 23.7 | 20.7384 | 3.94962 |
| 9 | 31.7 | 28.8476 | 3.72023 |
| 10 | 7.99 | 11.8315 | 3.72023 |
| 11 | 20.8 | 22.9481 | 3.62442 |
| 12 | 37.0 | 36.9568 | 3.94962 |
| 13 | 15.4 | 17.0487 | 3.72023 |
| 14 | 29.6 | 31.1729 | 3.94962 |
| 15 | 5.65 | 2.92454 | 3.94962 |
| 16 | 37.8 | 34.0648 | 3.72023 |
| 17 | 20.9 | 20.7384 | 3.94962 |
| 18 | 33.8 | 28.8476 | 3.72023 |
| 19 | 11.1 | 11.8315 | 3.72023 |
| 20 | 24.2 | 22.9481 | 3.62442 |
| 21 | 29.6 | 36.9568 | 3.94962 |
| 22 | 10.2 | 17.0487 | 3.72023 |
| 23 | 33.3 | 31.1729 | 3.94962 |
| 24 | 5.82 | 2.92454 | 3.94962 |
| 25 | 37.0 | 34.0648 | 3.72023 |
| 26 | 14.9 | 20.7384 | 3.94962 |
| 27 | 31.5 | 28.8476 | 3.72023 |

**Response optimisation**

Goal: maximise Zeta-potential

Optimal value = 36.9568 mV

| *Factor* | *Low* | *High* | *Optimal* |
| --- | --- | --- | --- |
| X_A_ | 0.0 | 2.5 | 0.0 |
| X_B_ | 0.25 | 1.25 | 0.25 |

**Factorial analysis of experiments for PDI**

**Initial model**

**Estimated effects for PDI**

| *Effect* | *Estimated* | *Std. Error* | *V.I.F.* |
| --- | --- | --- | --- |
| Mean | 0.27737 | 0.0169786 |  |
| X_A_ | -0.187889 | 0.0185992 | 1.0 |
| X_B_ | -0.111 | 0.0185992 | 1.0 |
| X_A_^2^ | 0.209222 | 0.0322147 | 1.0 |
| X_A,B_ | 0.1575 | 0.0227792 | 1.0 |
| X_B_^2^ | -0.0247778 | 0.0322147 | 1.0 |

Standard errors based on total error with 21 d.f.

**Analysis of Variance for PDI**

| *Source* | *Sum of squares* | *DF* | *Mean Square* | *F* | *P* |
| --- | --- | --- | --- | --- | --- |
| X_A_ | 0.15886 | 1 | 0.15886 | 102.05 | 0.0000 |
| X_B_ | 0.0554445 | 1 | 0.0554445 | 35.62 | 0.0000 |
| X_A_^2^ | 0.0656609 | 1 | 0.0656609 | 42.18 | 0.0000 |
| X_A,B_ | 0.0744188 | 1 | 0.0744188 | 47.81 | 0.0000 |
| X_B_^2^ | 0.000920907 | 1 | 0.000920907 | 0.59 | 0.4504 |
| Total Error | 0.0326903 | 21 | 0.00155668 |  |  |
| Total (corr.) | 0.387995 | 26 |  |  |  |

R^2^ = 91.5746 %

R^2^ _adj_ = 89.5685 %

Standard error of the estimate = 0.0394548

Mean absolute error = 0.0285391

Durbin-Watson = 2.22311 (P=0.7661)

Lag 1 residual autocorrelation = -0.140938

**Recalculated model**

**Estimated effects for PDI**

| *Effect* | *Estimated* | *Std. Error* | *V.I.F.* |
| --- | --- | --- | --- |
| Mean | 0.269111 | 0.0130289 |  |
| X_A_ | -0.187889 | 0.0184257 | 1.0 |
| X_B_ | -0.111 | 0.0184257 | 1.0 |
| X_A_^2^ | 0.209222 | 0.0319143 | 1.0 |
| X_A,B_ | 0.1575 | 0.0225668 | 1.0 |

Standard errors based on total error with 22 d.f.

**Analysis of Variance for PDI**

| *Source* | *Sum of squares* | *DF* | *Mean Square* | *F* | *P* |
| --- | --- | --- | --- | --- | --- |
| X_A_ | 0.15886 | 1 | 0.15886 | 103.98 | 0.0000 |
| X_B_ | 0.0554445 | 1 | 0.0554445 | 36.29 | 0.0000 |
| X_A_^2^ | 0.0656609 | 1 | 0.0656609 | 42.98 | 0.0000 |
| X_A,B_ | 0.0744188 | 1 | 0.0744188 | 48.71 | 0.0000 |
| Total Error | 0.0336112 | 22 | 0.00152778 |  |  |
| Total (corr.) | 0.387995 | 26 |  |  |  |

R^2^ = 91.3372 %

R^2^ _adj_ = 89.7622 %

Standard error of the estimate = 0.0390868

Mean absolute error = 0.0294568

Durbin-Watson = 2.27617 (P=0.8162)

Lag 1 residual autocorrelation = -0.168108

**Regression coefficients for PDI**

| *Coefficient* | *Estimated* |
| --- | --- |
| β_0_ | 0.669042 |
| β_A_ | -0.337033 |
| β_B_ | -0.2685 |
| β_A,A_ | 0.0669511 |
| β_A,B_ | 0.126 |

**Correlation Matrix for Estimated Effects**

|  |  | (1) | (2) | (3) | (4) | (5) |
| --- | --- | --- | --- | --- | --- | --- |
| (1) | Mean | 1.0000 | 0.0000 | 0.0000 | -0.8165 | 0.0000 |
| (2) | X_A_ | 0.0000 | 1.0000 | 0.0000 | 0.0000 | 0.0000 |
| (3) | X_B_ | 0.0000 | 0.0000 | 1.0000 | 0.0000 | 0.0000 |
| (4) | X_A_^2^ | -0.8165 | 0.0000 | 0.0000 | 1.0000 | 0.0000 |
| (5) | X_A,B_ | 0.0000 | 0.0000 | 0.0000 | 0.0000 | 1.0000 |

**Estimated Results for PDI**

| *Run* | *Observed values* | *Adjusted values* | *Std. Error* |
| --- | --- | --- | --- |
| 1 | 0.269 | 0.279778 | 0.0412012 |
| 2 | 0.267 | 0.269111 | 0.0412012 |
| 3 | 0.677 | 0.601917 | 0.0437004 |
| 4 | 0.266 | 0.213611 | 0.0422186 |
| 5 | 0.315 | 0.333417 | 0.0437004 |
| 6 | 0.283 | 0.303028 | 0.0437004 |
| 7 | 0.464 | 0.467667 | 0.0412012 |
| 8 | 0.283 | 0.256528 | 0.0437004 |
| 9 | 0.283 | 0.324611 | 0.0422186 |
| 10 | 0.303 | 0.279778 | 0.0412012 |
| 11 | 0.279 | 0.269111 | 0.0412012 |
| 12 | 0.583 | 0.601917 | 0.0437004 |
| 13 | 0.267 | 0.213611 | 0.0422186 |
| 14 | 0.275 | 0.333417 | 0.0437004 |
| 15 | 0.282 | 0.303028 | 0.0437004 |
| 16 | 0.499 | 0.467667 | 0.0412012 |
| 17 | 0.267 | 0.256528 | 0.0437004 |
| 18 | 0.261 | 0.324611 | 0.0422186 |
| 19 | 0.29 | 0.279778 | 0.0412012 |
| 20 | 0.256 | 0.269111 | 0.0412012 |
| 21 | 0.593 | 0.601917 | 0.0437004 |
| 22 | 0.262 | 0.213611 | 0.0422186 |
| 23 | 0.306 | 0.333417 | 0.0437004 |
| 24 | 0.257 | 0.303028 | 0.0437004 |
| 25 | 0.497 | 0.467667 | 0.0412012 |
| 26 | 0.284 | 0.256528 | 0.0437004 |
| 27 | 0.281 | 0.324611 | 0.0422186 |

**Response optimisation**

Goal: minimise PDI

Optimal value = 0.213059

| *Factor* | *Low* | *High* | *Optimal* |
| --- | --- | --- | --- |
| X_A_ | 0.0 | 2.5 | 1.3408 |
| X_B_ | 0.25 | 1.25 | 1.25 |

**Factorial analysis of experiments for EE**

**Initial model**

**Estimated effects for EE (%)**

| *Effect* | *Estimated* | *Std. Error* | *V.I.F.* |
| --- | --- | --- | --- |
| Mean | 99.5185 | 0.0737755 |  |
| X_A_ | 0.301222 | 0.080817 | 1.0 |
| X_B_ | 0.979444 | 0.080817 | 1.0 |
| X_A_^2^ | -0.110111 | 0.139979 | 1.0 |
| X_A,B_ | -0.4275 | 0.0989802 | 1.0 |
| X_B_^2^ | -0.652778 | 0.139979 | 1.0 |

Standard errors based on total error with 21 d.f.

**Analysis of Variance for EE**

| *Source* | *Sum of squares* | *DF* | *Mean Square* | *F* | *P* |
| --- | --- | --- | --- | --- | --- |
| X_A_ | 0.408307 | 1 | 0.408307 | 13.89 | 0.0012 |
| X_B_ | 4.3169 | 1 | 4.3169 | 146.88 | 0.0000 |
| X_A_^2^ | 0.0181867 | 1 | 0.0181867 | 0.62 | 0.4403 |
| X_A,B_ | 0.548269 | 1 | 0.548269 | 18.65 | 0.0003 |
| X_B_^2^ | 0.639178 | 1 | 0.639178 | 21.75 | 0.0001 |
| Total Error | 0.617216 | 21 | 0.0293913 |  |  |
| Total (corr.) | 6.54806 | 26 |  |  |  |

R^2^ = 90.5741 %

R^2^ _adj_ = 88.3298 %

Standard error of the estimate = 0.171439

Mean absolute error = 0.114995

Durbin-Watson = 1.84983 (P=0.4187)

Lag 1 residual autocorrelation = -0.0722663

**Recalculated model**

**Estimated effects for EE (%)**

| *Effect* | *Estimated* | *Std. Error* | *V.I.F.* |
| --- | --- | --- | --- |
| Mean | 99.4818 | 0.056649 |  |
| X_A_ | 0.301222 | 0.0801137 | 1.0 |
| X_B_ | 0.979444 | 0.0801137 | 1.0 |
| X_A_,_B_ | -0.4275 | 0.0981189 | 1.0 |
| X_B_^2^ | -0.652778 | 0.138761 | 1.0 |

Standard errors based on total error with 22 d.f.

**Analysis of Variance for EE**

| *Source* | *Sum of squares* | *DF* | *Mean Square* | *F* | *P* |
| --- | --- | --- | --- | --- | --- |
| X_A_ | 0.408307 | 1 | 0.408307 | 14.14 | 0.0011 |
| X_B_ | 4.3169 | 1 | 4.3169 | 149.47 | 0.0000 |
| X_A_,_B_ | 0.548269 | 1 | 0.548269 | 18.98 | 0.0003 |
| X_B_^2^ | 0.639178 | 1 | 0.639178 | 22.13 | 0.0001 |
| Total Error | 0.635403 | 22 | 0.028882 |  |  |
| Total (corr.) | 6.54806 | 26 |  |  |  |

R^2^ = 90.2963 %

R^2^ _adj_ = 88.532 %

Standard error of the estimate = 0.169947

Mean absolute error = 0.119292

Durbin-Watson = 2.17362 (P=0.6900)

Lag 1 residual autocorrelation = -0.206929

**Correlation Matrix for Estimated Effects**

| *Coefficient* | *Estimated* |
| --- | --- |
| β_0_ | 97.5416 |
| β_A_ | 0.376989 |
| β_B_ | 3.36528 |
| β_A,B_ | -0.342 |
| β_B,B_ | -1.30556 |

**Correlation Matrix for Estimated Effects**

|  |  | (1) | (2) | (3) | (4) | (5) |
| --- | --- | --- | --- | --- | --- | --- |
| (1) | Mean | 1.0000 | 0.0000 | 0.0000 | 0.0000 | -0.8165 |
| (2) | X_A_ | 0.0000 | 1.0000 | 0.0000 | 0.0000 | 0.0000 |
| (3) | X_B_ | 0.0000 | 0.0000 | 1.0000 | 0.0000 | 0.0000 |
| (4) | X_A_,_B_ | 0.0000 | 0.0000 | 0.0000 | 1.0000 | 0.0000 |
| (5) | X_B_^2^ | -0.8165 | 0.0000 | 0.0000 | 0.0000 | 1.0000 |

**Estimated Results for EE**

| *Run* | *Observed values* | *Adjusted values* | *Std. Error* |
| --- | --- | --- | --- |
| 1 | 99.604 | 99.6324 | 0.183564 |
| 2 | 99.624 | 99.4818 | 0.17914 |
| 3 | 98.155 | 98.3013 | 0.190006 |
| 4 | 99.753 | 99.6451 | 0.17914 |
| 5 | 99.528 | 99.7083 | 0.190006 |
| 6 | 99.599 | 99.582 | 0.190006 |
| 7 | 99.4 | 99.3312 | 0.183564 |
| 8 | 99.021 | 99.03 | 0.190006 |
| 9 | 99.004 | 98.6657 | 0.17914 |
| 10 | 99.344 | 99.6324 | 0.183564 |
| 11 | 99.582 | 99.4818 | 0.17914 |
| 12 | 98.277 | 98.3013 | 0.190006 |
| 13 | 99.719 | 99.6451 | 0.17914 |
| 14 | 99.67 | 99.7083 | 0.190006 |
| 15 | 99.69 | 99.582 | 0.190006 |
| 16 | 99.458 | 99.3312 | 0.183564 |
| 17 | 99.2 | 99.03 | 0.190006 |
| 18 | 98.829 | 98.6657 | 0.17914 |
| 19 | 99.438 | 99.6324 | 0.183564 |
| 20 | 99.43 | 99.4818 | 0.17914 |
| 21 | 98.208 | 98.3013 | 0.190006 |
| 22 | 99.491 | 99.6451 | 0.17914 |
| 23 | 99.705 | 99.7083 | 0.190006 |
| 24 | 99.651 | 99.582 | 0.190006 |
| 25 | 99.456 | 99.3312 | 0.183564 |
| 26 | 99.021 | 99.03 | 0.190006 |
| 27 | 98.276 | 98.6657 | 0.17914 |

**Response optimisation**

Goal: maximise EE

Optimal value = 99.7083%

| *Factor* | *Low* | *High* | *Optimal* |
| --- | --- | --- | --- |
| X_A_ | 0.0 | 2.5 | 0.0 |
| X_B_ | 0.25 | 1.25 | 1.25 |

**Multiple Response Optimization**

Responses:

Z-average diameter (nm)

PDI

Zeta-potential (mV)

| Response | *Lower observed* | *Higher observed* |
| --- | --- | --- |
| Z-average diameter | 617.0 | 2292.0 |
| PDI | 0.256 | 0.677 |
| Zeta-potential | 5.54 | 37.8 |

| *Response* | *Desirability* | | *Goal* | *Weight* | *Impact* |
| --- | --- | --- | --- | --- | --- |
|  | *Low* | *High* |  |  |  |
| Z-average diameter | 400.0 | 1000.0 | Minimise | 2.0 | 3.0 |
| PDI | 0.256 | 0.677 | Minimise | 1.0 | 3.0 |
| Zeta-potential | 5.54 | 37.8 | Maximise | 1.0 | 3.0 |

| *Run* | *Z-average diameter* | *PDI* | *Zeta-potential* | *Desirability* | |
| --- | --- | --- | --- | --- | --- |
|  |  |  |  | *Expected* | *Observed* |
| 1 | 746.0 | 0.269 | 12.6 | 0.347973 | 0.336223 |
| 2 | 659.0 | 0.267 | 21.4 | 0.55705 | 0.536763 |
| 3 | 893.0 | 0.677 | 34.5 | 0.200726 | 0.0 |
| 4 | 653.0 | 0.266 | 14.1 | 0.192401 | 0.442495 |
| 5 | 2139.0 | 0.315 | 31.8 | 0.0 | 0.0 |
| 6 | 712.0 | 0.283 | 5.54 | 0.0 | 0.0 |
| 7 | 1355.0 | 0.464 | 37.7 | 0.0 | 0.0 |
| 8 | 623.0 | 0.283 | 23.7 | 0.374442 | 0.592491 |
| 9 | 629.0 | 0.283 | 31.7 | 0.845651 | 0.66203 |
| 10 | 702.0 | 0.303 | 7.99 | 0.347973 | 0.255313 |
| 11 | 665.0 | 0.279 | 20.8 | 0.55705 | 0.518513 |
| 12 | 686.0 | 0.583 | 37.0 | 0.200726 | 0.39069 |
| 13 | 684.0 | 0.267 | 15.4 | 0.192401 | 0.43544 |
| 14 | 2237.0 | 0.275 | 29.6 | 0.0 | 0.0 |
| 15 | 751.0 | 0.282 | 5.65 | 0.0 | 0.081981 |
| 16 | 1703.0 | 0.499 | 37.8 | 0.0 | 0.0 |
| 17 | 680.0 | 0.267 | 20.9 | 0.374442 | 0.509028 |
| 18 | 617.0 | 0.261 | 33.8 | 0.845651 | 0.706542 |
| 19 | 738.0 | 0.29 | 11.1 | 0.347973 | 0.311444 |
| 20 | 666.0 | 0.256 | 24.2 | 0.55705 | 0.563827 |
| 21 | 744.0 | 0.593 | 29.6 | 0.200726 | 0.300332 |
| 22 | 694.0 | 0.262 | 10.2 | 0.192401 | 0.333331 |
| 23 | 2292.0 | 0.306 | 33.3 | 0.0 | 0.0 |
| 24 | 743.0 | 0.257 | 5.82 | 0.0 | 0.116683 |
| 25 | 1250.0 | 0.497 | 37.0 | 0.0 | 0.0 |
| 26 | 721.0 | 0.284 | 14.9 | 0.374442 | 0.388337 |
| 27 | 628.0 | 0.281 | 31.5 | 0.845651 | 0.662642 |

**Desirability optimisation**

Optimal value = 0.848795

| *Factor* | *Low* | *High* | *Optimal* |
| --- | --- | --- | --- |
| X_A_ | 0.0 | 2.5 | 1.4455 |
| X_B_ | 0.25 | 1.25 | 0.25 |

| *Response* | *Optimal* |
| --- | --- |
| Z-average diameter | 400.0 |
| PDI | 0.30016 |
| Zeta-potential | 27.5793 |
